# Establishment of Epidemiological Resistance Cut-Off Values of Aquatic *Aeromonas* to Eight Antimicrobial Agents

**DOI:** 10.3390/microorganisms10040776

**Published:** 2022-04-05

**Authors:** Yaoyao Lin, Jicheng Yang, Zhenbing Wu, Qianqian Zhang, Shuyi Wang, Jingwen Hao, Lijian Ouyang, Aihua Li

**Affiliations:** 1 State Key Laboratory of Freshwater Ecology and Biotechnology, Institute of Hydrobiology, Chinese Academy of Sciences, Wuhan 430072, China; linyaoyao@ihb.ac.cn (Y.L.); yangjc@ihb.ac.cn (J.Y.); wuzhenbing@ihb.ac.cn (Z.W.); zqq@ihb.ac.cn (Q.Z.); wangsy@ihb.ac.cn (S.W.); haojingwen@ihb.ac.cn (J.H.); 2 University of Chinese Academy of Sciences, Beijing 100049, China; 3 College of Fisheries and Life, Dalian Ocean University, Dalian 116023, China; 4 School of Environmental Science and Engineering, Huazhong University of Science and Technology, Wuhan 430074, China; 5 Key Laboratory of Aquaculture Disease Control, Ministry of Agriculture, Wuhan 430072, China; 6 National Aquatic Biological Resource Center (NABRC), Wuhan 430072, China; 7 Ecological Engineering College, Guizhou University of Engineering Science, Bijie 551700, China

**Keywords:** *Aeromonas*, epidemiological cut-off values, resistance rate, MIC_50_, MIC_90_

## Abstract

The abuse of antibiotics in aquaculture has led to the increasing rate of antibiotic resistance of aquatic bacteria including *Aeromonas*, which is an increasing threat to environmental and human health. To date, no epidemiological cut-off values (CO_WT_) for *Aeromonas* spp. have been established by the Clinical and Laboratory Standards Institute nor the European Commission on Antimicrobial Susceptibility Testing. In this study, commercially prepared minimum inhibitory concentration (MIC) test 96-well plates (dry-form plates) were used to determine the MIC of eight antimicrobial agents against 556 *Aeromonas* strains. The obtained MIC distributions were simulated and analyzed by NRI and ECOFFinder to obtain tentative CO_WT_ values for *Aeromonas* spp. The CO_WT_ values of eight kinds of representative antimicrobial agents including trimethoprim–sulfamethoxazole, erythromycin, doxycycline, neomycin, colistin, florfenicol, enrofloxacin, and ceftazidime for *Aeromonas* spp. were established and were 0.25, 64/32, 4/2, 8, 4, 1, 0.062/0.125, and 0.5 μg/mL, respectively. Results showed that *Aeromonas* spp. had a very high proportion of non-wild-type strains to enrofloxacin, florfenicol, and doxycycline, which are the most widely used antimicrobials in aquaculture. The CO_WT_ values for *Aeromonas* spp. obtained in this study can contribute to the final establishment of CO_WT_ for *Aeromonas* spp. internationally.

## 1. Introduction

Antimicrobial resistance (AMR) has become a growing threat to global public health [1]. The continuous use of antibiotics has led to the emergence of drug resistance in some bacteria. Due to the difficulty of clinical treatment and high mortality, it poses a huge threat to human health [2]. According to the World Health Organization, antibiotic resistance is one of the top 10 global health threats and one of the major global health challenges in the 21st century [3]. Every year, 700,000 people die from drug-resistant bacterial infections around the world. It is estimated that by 2050, bacterial resistance will cause 10 million deaths worldwide each year [4].

The aquatic environment may provide an ideal environment for the acquisition and spread of antibiotic resistance because it is closely related to human activities [5], such as aquaculture, wastewater discharge, hydraulic engineering, and agricultural measures. Recent studies have also shown that aquatic ecosystems are important reservoirs of antibiotic resistance genes and resistant bacteria, one of the main ways in which resistant bacteria are transmitted from animals to humans, and a potential channel for the transmission of human pathogens [6]. *Aeromonas* is a Gram-negative facultative anaerobic bacillus widely distributed in aquatic ecosystems, which can infect not only fish and other aquatic animals but also humans and livestock. It is an opportunistic pathogen of human–fish–animal symbiosis. *Aeromonas* not only causes a wide range of diseases in humans such as bacteremia/septicemia, gastroenteritis, wound infection, and infection immunodeficiency. Some diseases caused in fish also seriously restrict the healthy development of the aquatic animal breeding industry [7]. *Aeromonas* can acquire antibiotic resistance mechanisms and has the potential for horizontal gene transfer, so it may be a good candidate for tracking the spread of antibiotic resistance in the water [8]. At present, *Aeromonas* has been accepted as an indicator organism for monitoring water quality and sewage pollution, and it is suitable for evaluating the prevalence, development, and spread of AMR in the aquatic environment [9].

To determine whether bacteria are susceptible to antimicrobial drugs, a drug resistance criterion needs to be established, that is, a breakpoint, a specific inhibition zone diameter, or a minimum inhibitory concentration (MIC) value. If there is no drug resistance criterion, drug resistance cannot be measured. The purpose of establishing the drug resistance criterion is to evaluate the status of bacterial drug resistance, guide clinical medication, and assist clinical treatment [10]. At present, many organizations establish breakpoints, but the widely used reference for drug breakpoints is the Clinical and Laboratory Standards Institute (CLSI) in the United States and European Commission on Antimicrobial Susceptibility Testing (EUCAST) in the European Union. The breakpoints developed by CLSI and EUCAST are roughly the same and are mainly divided into wild-type cut-off (CO_WT_)/epidemiological cut-off values (ECOFF) and clinical breakpoints (CBPs). The main purpose of the establishment of CO_WT_/ECOFF is to epidemiologically monitor AMR phenotypes, investigate whether bacteria are sensitive or resistant to antimicrobial drugs and distinguish bacteria into wild type (WT) and non-wild type (NWT), and clinical effects are not considered. CBPs are mainly used to distinguish between good prognosis and treatment failure bacteria, which is obtained by comparing the clinical treatment effect of different MIC pathogens [11].

The main method to formulate CO_WT_ values is to conduct an antimicrobial susceptibility test (AST) and then perform statistical fitting analysis on the AST results. CLSI and EUCAST have published AST methods including micro-/macro-broth dilution method, agar dilution method, and disk diffusion method. The micro-broth dilution method is currently the most commonly used method for aquatic bacteria. It quantitatively obtains the MICs of antimicrobials on bacteria by setting a two-fold serial dilution of the drug concentration gradient [12,13,14,15]. AST results are divided into sensitive (S), intermediate (I), and resistant (R) [16].

At present, there are very few reference standards and studies for CBPs for *Aeromonas*. CLSI [17] only provides the MIC or zone diameter CBPs for *A. salmonicida* to oxytetracycline and oxolinic acid and the epidemiological cut-off values to seven drugs including gentamicin, erythromycin, florfenicol, ormetoprim–sulfadimethoxine, trimethoprim–sulfamethoxazole, oxytetracycline, and oxolinic acid. It also provides the epidemiological cut-off values for *A. hydrophila* to six drugs including gentamicin, erythromycin, florfenicol, oxytetracycline, enrofloxacin, and oxolinic acid. The only MIC or zone diameter breakpoints established by EUCAST [18] are the CBPs for *Aeromonas* spp. to six drugs including cefepime, ceftazidime, aztreonam, ciprofloxacin, levofloxacin, and trimethoprim–sulfamethoxazole. Most of these antimicrobials are not commonly used in aquaculture, so the reference values of these CBPs or CO_WT_ values are limited. Literature [12] reported the MIC tentative CO_WT_ values for *A. bestiarum*, *A. salmonicida*, *A. sobria*, and *A. eucrenophila* and *Aeromonas* spp. against 15 antimicrobials such as florfenicol, colistin, and enrofloxacin. However, these results have not been verified by other studies.

China is the most important country in the world for aquaculture, with high aquaculture production and complex use of antimicrobial drugs [19]. Although many scholars have investigated the AMR status of fish pathogenic bacteria in China, especially the pathogenic *Aeromonas*, the CBPs used in these studies mainly referred to ones of CLSI, which are unsuitable for the clinical treatment of fish diseases nor suitable for the analysis of the prevalence of AMR in aquaculture. And whether the CO_WT_ values established in the above literature [12] are equally applicable to water bodies and the aquaculture environment in China needs to be verified.

Therefore, this study aimed to establish CO_WT_ criteria of *Aeromonas* spp. to investigate and analyze the current AMR status of *Aeromonas* spp. in typical aquaculture ponds, a reservoir, and a natural river in China.

## 2. Materials and Methods

### 2.1. Isolation and Identification of Bacteria

From November 2020 to October 2021, sick and healthy fish, water, or sediment samples from fish ponds in Jingzhou City, Hubei Province; sick and healthy fish samples from fish ponds in Wuhan City, Hubei Province, and Xiashan reservoir in Weifang City, Shandong Province; and healthy fish samples from Liuchong River in Bijie City, Guizhou Province, were collected and transported to the laboratory through ice boxes to perform bacterial isolation. The clinical symptoms of the sick fish were swirling on the surface of the water body, multiple congestions on the body surface, body ulceration; yellowing of the body, increased mucus, swelling of the abdomen with ascites; split head, khaki liver; ulcerative erythema on the body surface; anal swelling and gently press on the abdomen that had yellow mucus or blood coming out of the anus, with a shriveled gut and congested gut walls. And fish without any of the above symptoms was judged as healthy fish. The intestinal, kidney, liver, spleen, brain, or blood of the sampled fish were streaked onto sterile ampicillin MacConkey agar (i.e., *Aeromonas* selection medium). The water sample or sediment sample diluted with 0.86% sterile normal saline was pipetted onto the sterile ampicillin MacConkey agar and spread evenly with a triangular glass rod, and all plates were incubated at 28 °C for 12–24 h. The pink single colonies were picked and cultivated overnight in a 1 mL BHI broth medium for bacterial identification.

The above bacterial suspension was used as the DNA template. The bacterial universal primer pair 27F (5′-AGAGTTTGATCCTGGCTCAG-3′) and 1492R (5′-GGTTACCTTGTTACGACTT-3′) was used for 16S rRNA gene PCR amplification. Amplification conditions were as follows: 94 °C for 5 min at pretreatment; 30 cycles of 94 °C for 30 s, 55 °C for 30 s, 72 °C for 90 s, and final extension of 72 °C for 7 min. The PCR products were sent to Wuhan Aikangjian Biotechnology Co., Ltd. (Wuhan China) for sequencing. Sequence homology analysis (bacterial identification) was performed using the Nucleotide BLAST in NCBI (https://www.ncbi.nlm.nih.gov/) (accessed on 8 February 2022). The obtained *Aeromonas* bacterial suspensions were then stored in a −80 °C refrigerator with 20% glycerol.

### 2.2. Determination of MICs

To ensure the accuracy and reproducibility of the experiment, a commercial 96-well MIC assay plate (Tianjin Jinzhang Science and Technology Development Co., Ltd., Tianjin, China) was used in this study. The assay plate contained lyophilized powder of the drug, and a two-fold dilution method was used to prepare the drug concentration gradient. The assay plates were manufactured in full compliance with the relevant CLSI protocols [20,21], and quality control and quality inspection were performed at the factory. Eight test drugs were set up in each assay plate, and their names, category, and concentration ranges were as follows: trimethoprim–sulfamethoxazole (TMP/SMZ) (sulfonamides) (0.062/1.187–64/1216 μg/mL), erythromycin (macrolides) (0.125–256 μg/mL), doxycycline (tetracyclines) (0.062–128 μg/mL), neomycin (aminoglycosides) (0.062–256 μg/mL), colistin (poly-peptides) (0.062–256 μg/mL), florfenicol (chloramphenicol) (0.062–256 μg/mL), enrofloxacin (fluoroquinolones) (0.008–64 μg/mL), and ceftazidime (cephalosporins) (0.016–128 μg/mL). These drugs include the most commonly used species in Chinese aquaculture, as well as medically important antimicrobials and antibiotics of different structural classes. The species selected for this study have a lot of crossover with those tested in other literature for comparison [12,22,23,24,25,26,27].

The MIC was determined according to the instructions of the MIC assay plate. The specific method is as follows: pick fresh single colonies suspended in cation-adjusted Mueller–Hinton broth, adjust the concentration of the bacterial solution to 0.5 McFarland concentration, and then dilute at a ratio of 1:200 so that the inoculum concentration of the bacterial solution is about 5 × 10^5^ CFU/mL. About 100 μL of this bacterial solution was added to each well of the MIC assay plate, and positive and negative controls were set up simultaneously. The plates were incubated at 28 °C for 24–28 h, and then MIC values were recorded. The quality control strain used was ATCC 25922, and the results were considered valid only if the MIC of the quality control strain was within the specified quality control range.

### 2.3. Determination of Tentative Epidemiological Cut-Off Values (CO_WT_)

CO_WT_ values were determined using the free and automated EXCEL spreadsheet programs ECOFFinder (available online: https://clsi.org/meetings/microbiology/ecoffinder/) and NRI (available online: http://www.bioscand.se/nri/) (both were accessed on 11 February 2022). These two methods were proposed by Turnidge et al. [28] and Kronvall [29], respectively, which will be referred to as Turnidge method and Kronvall method hereinafter. The principle was to use the method of nonlinear regression analysis to fit the cumulative distribution of MICs, inputting the distribution of MICs according to the operation instructions, and the program will automatically fit and calculate the CO_WT_ values of bacteria. The NRI program directly provides CO_WT_ values, whereas the ECOFFinder program selects the MICs with a confidence interval of 95.0% or 97.5% as the final CO_WT_ values. ECV and EOCFF are used for epidemiological cut-off values set by two international institutions, CLSI and EUCAST, respectively. To avoid confusion, the epidemiological cut-off values are abbreviated as CO_WT_ in this study.

### 2.4. Calculation of MIC_50_, MIC_90_, and Resistance Rate (NWT%)

MIC_50_ is the MIC required to inhibit 50% bacterial growth, and MIC_90_ is the MIC required to inhibit 90% bacterial growth. The strains with MIC values greater than the breakpoint value of the 95% or 97.5% confidence interval are all NWT strains. The strains with MIC values less than or equal to the breakpoint value of the 95% or 97.5% confidence interval are WT strains. The resistance rate is equal to the ratio of the number of NWT strains to the total number of strains tested in the present study.

## 3. Results

### 3.1. Aeromonas Diversity

**Figure 1:**
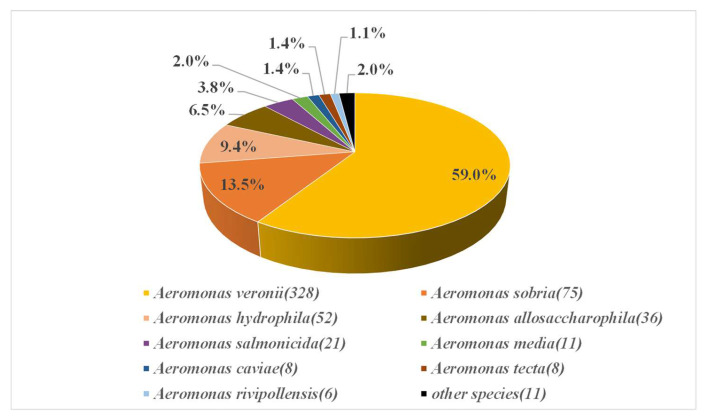
Diversity and proportion of 556 Aeromonas strains. Numbers in parentheses indicate the number of strains, other species correspond to *A. encheleia* (5) *A. popoffii* (3) *A. dhakensis* (1) *A. enteropelogenes* (1), and *A. jandaei* (1).

A total of 556 strains of *Aeromonas* were assayed for their MIC, and the proportion of strains in four sampling areas was as follows: 369 strains in Jingzhou City, Hubei Province; 30 strains in Wuhan City, Hubei Province; 16 strains in Weifang City, Shandong Province; and 141 strains in Bijie City, Guizhou Province. The 556 *Aeromonas* strains were derived from 183 fish, 14 water samples, and 5 sediment samples. The specific source and quantity distribution of the strains are shown in Table 1. Among them, 14 species were identified at the species level, and the proportions are shown in Figure 1. Among them, *A. veronii* (*n* = 328, 59.0%), *A. sobria* (*n* = 75, 13.5%), *A. hydrophila* (*n* = 52, 9.4%), and *A. allosaccharophila* (*n* = 36, 6.5%) were very abundant, accounting for 88.3% of the total number of *Aeromonas*.

### 3.2. MIC Measurement Results

A total of four batches of MIC assay plates were used in this study. Quality control tests were performed for each batch using the standard strain ATCC 25922, and the results were all within the quality control range except for neomycin and erythromycin, for which no quality control range was available. The MIC distributions of 556 *Aeromonas* strains to eight drugs are shown in Table 2, and the MICs of seven drugs exceeded the dilution range of the drugs. Drugs with MIC lower than or equal to the lowest concentration determined included trimethoprim–sulfamethoxazole (≤0.062, *n* = 212), florfenicol (≤0.125, *n* = 11), enrofloxacin (≤0.031, *n* = 99), and ceftazidime (≤0.062, *n* = 56). Drugs with MIC higher than the highest concentration measured were trimethoprim–sulfamethoxazole, erythromycin, neomycin, colistin, florfenicol, and ceftazidime. Only one drug, doxycycline, had MICs that were always within the drug dilution range.

### 3.3. Establishment of Tentative Epidemiological Cut-Off Values (CO_WT_)

**Figure 2:**
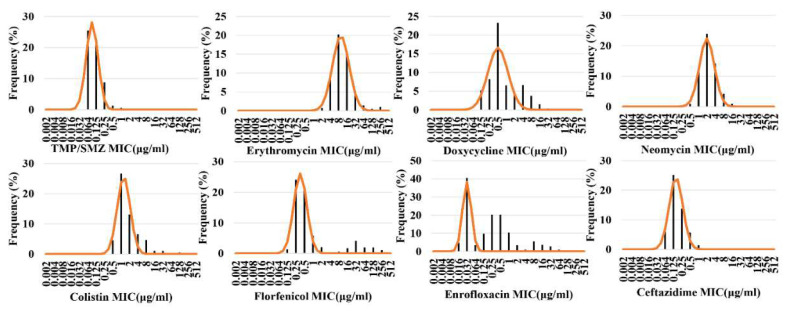
Nonlinear regression fitting distribution of eight drugs to *Aeromonas* spp. (Kronvall method).

**Figure 3:**
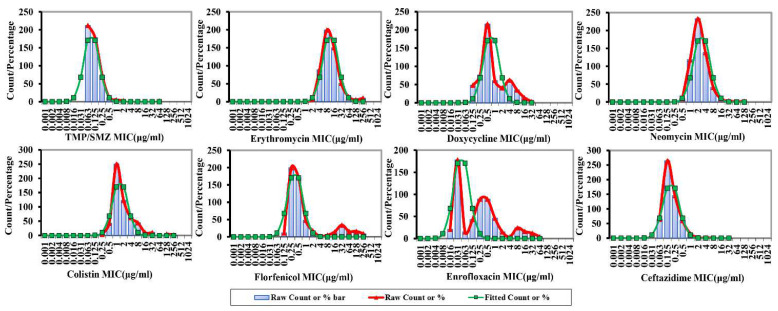
Nonlinear regression fitting distribution of eight drugs to *Aeromonas* spp. (Turnidge method). (Red “Raw Count or %” represents measured MIC data, green “Fitted Count or %” represents simulated MIC data).

The Kronvall and Turnidge method nonlinear regression fitting distributions of eight drugs against *Aeromonas* spp. are shown in Figure 2 and Figure 3, respectively. As can be seen, the nonlinear regression fitting distribution results of the eight drugs against *Aeromonas* spp. obtained by the Kronvall and Turnidge methods were almost identical. The MIC distribution of enrofloxacin against *Aeromonas* spp. was very wide and multimodal, with two peaks, one large and one small, on the right side. The MICs for doxycycline and florfenicol were widely distributed and showed no obvious bimodal distribution. The MIC distributions for the remaining five drugs were narrow and unimodal. The CO_WT_ values can be preliminarily observed with the naked eye, which is the upper limit of the 95% or 97.5% confidence interval of the abscissa of the fitting curve. Erythromycin had the largest CO_WT_ value, followed by neomycin and colistin, doxycycline, florfenicol, ceftazidime, trimethoprim–sulfamethoxazole, and enrofloxacin.

The CO_WT_, MIC_50_, and MIC_90_ (μg/mL) and NWT (%) of 556 *Aeromonas* strains against eight drugs are shown in Table 3. The CO_WT_ of *Aeromonas* spp. against each drug obtained by the NRI or ECOFFinder table processing program was consistent with that observed with the naked eye. The CO_WT_ values calculated by the Kronvall and Turnidge methods were the same for the five drugs, including trimethoprim–sulfamethoxazole, neomycin, colistin, florfenicol, and ceftazidime. The CO_WT_ values calculated by the two methods for the other three drugs had only a difference of one step of drug dilution concentration. The similarity rate of CO_WT_ obtained by the two methods could reach 62.5%. Using the CO_WT_ established in this study to calculate the number (percentage) of NWT strains among all strains collected in the present study, NWT (%), it could be concluded that enrofloxacin had the highest proportion of NWT strains of *Aeromonas*, exceeding 50%, followed by florfenicol. The two drugs with lower proportions were ceftazidime and neomycin. The difference between the MIC_50_ and MIC_90_ of trimethoprim–sulfamethoxazole was the largest, and its MIC_90_ exceeds the dilution range of the drug. The difference between the MIC_50_ and MIC_90_ of neomycin and ceftazidime was the smallest, with only two drug dilution gradients. The drug with the largest MIC_50_ and MIC_90_ was erythromycin, followed by enrofloxacin and doxycycline, and ceftazidime.

The CO_WT_, MIC_50_, and MIC_90_ (μg/mL) of the four *Aeromonas* spp. to the eight drugs are shown in Table 4. The CO_WT_ of *A. veronii* was the closest to that of *Aeromonas* spp., followed by *A. sobria*. The CO_WT_ of the four *Aeromonas* spp. differed for the eight drugs. *A. veronii* obtained the highest CO_WT_ similarity rate by Kronvall and Turnidge methods (up to 87.5%), and *A. allosaccharophila* obtained the lowest CO_WT_ similarity rate by the two methods. The largest difference between MIC_50_ and MIC_90_ was trimethoprim–sulfamethoxazole of *A. veronii*, and the smallest difference was trimethoprim–sulfamethoxazole of *A. sobria*. The MIC_50_ and MIC_90_ of eight drugs against *A. sobria* were smaller. For these four *Aeromonas* spp., the MIC_50_ of erythromycin was relatively high, whereas those of trimethoprim–sulfamethoxazole, ceftazidime, and enrofloxacin were relatively low.

### 3.4. Comparative Analysis of the Resistance Rate of Aeromonas spp. in Different Regions

**Figure 4:**
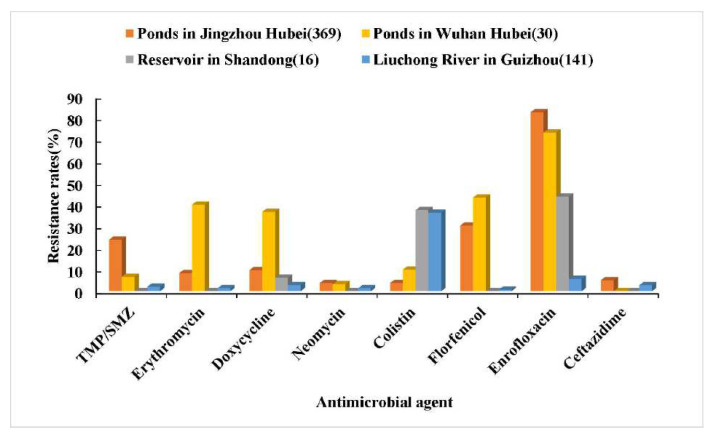
Comparative analysis of the resistance rates of *Aeromonas* spp. to eight drugs in different regions (CO_WT_ K). The numbers in parentheses represent the number of strains.

**Figure 5:**
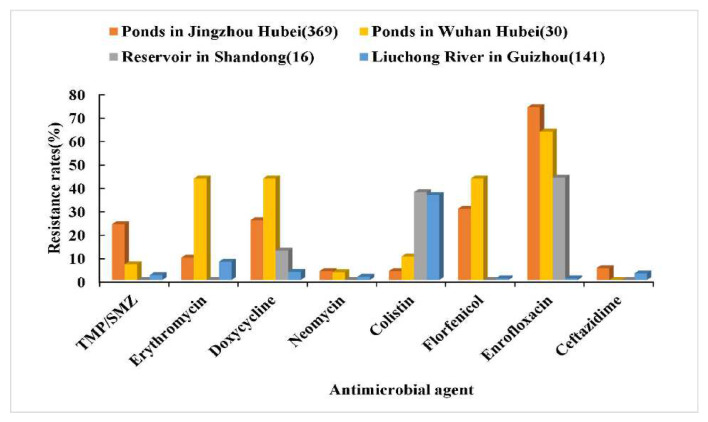
Comparative analysis of the resistance rates of *Aeromonas* spp. to eight drugs in different regions (CO_WT_ T). The numbers in parentheses represent the number of strains.

Using the above obtained CO_WT_ K and CO_WT_ T of *Aeromonas* spp. against eight drugs, the resistance rate was calculated according to the MIC distributions of *Aeromonas* spp. against eight drugs in each region. The results are shown in Figure 4 and Figure 5. *Aeromonas* spp. in culture ponds in Jingzhou City, Hubei Province, had high resistance rates to enrofloxacin, florfenicol, and trimethoprim–sulfamethoxazole. *Aeromonas* spp. in culture ponds in Wuhan City, Hubei Province, had high resistance rates to enrofloxacin, florfenicol, erythromycin, and doxycycline, but zero resistance to ceftazidime. *Aeromonas* spp. in the two ponds had the highest resistance rate to enrofloxacin (up to 82.7%), followed by florfenicol. The resistance rates of *Aeromonas* spp. in Xiashan Reservoir of Shandong Province to five drugs including trimethoprim–sulfamethoxazole, erythromycin, neomycin, florfenicol, and ceftazidime were 0, but the resistance rates of *Aeromonas* spp. to enrofloxacin and colistin were high. *Aeromonas* spp. in Liuchong River, Guizhou Province, had a high resistance rate to colistin, but their resistance rates to the other seven drugs were all low.

## 4. Discussion

The establishment of resistance breakpoints is influenced by different laboratories in terms of assay protocols, reagents, and human factors, and the results of the established breakpoints may vary greatly. Therefore, only the breakpoints established following the standard experimental protocols, such as that of CLSI, can be widely accepted and recognized. Many researchers have used *Aeromonas* as indicator bacteria for antimicrobial susceptibility in aquatic environments [8,30,31]. *Aeromonas* spp. are ubiquitous and pathogenic to many aquatic animals and can be isolated in freshwater systems at any time of the year [8]. *A. hydrophila*, *A. veronii*, *A. sobria*, and many other *Aeromonas* species are the most common pathogenic bacteria in aquaculture in China and have a negative impact on the Chinese aquaculture industry [32]. Therefore, we chose *Aeromonas* spp. as the first aquatic bacterial species to establish the resistance threshold. This is the first study of this type conducted using Chinese isolates.

In this study, *Aeromonas* were isolated from fish collected from a natural non-aquaculture river and aquaculture ponds in China. The bacterial strains from these two sources are defined as environmental or pathogen strains. The protocols reported in the literature [12] were used to establish the CO_WT_ values for *Aeromonas*. The results obtained in the present study can be used for comparison with those reported in the literature [12] to verify the reproducibility of the method and to determine whether CO_WT_ values are influenced by the geographical origin of the bacterial strains. Additionally, it can provide breakpoints for determining the epidemiological characteristics of *Aeromonas* AMR to antimicrobials of greatest concern to the Chinese aquaculture industry. Commercially prepared MIC test 96-well plates containing dehydrated antimicrobial agents (dry-form plates) were used in this study, this standardized method made the experimental results more accurate and reproducible. During the actual operation with NRI and ECOFFinder, the MICs of the 95% and 97.5% confidence intervals of ECOFFinder were the same, and most of the CO_WT_ values obtained by selecting the 95% or 97.5% confidence intervals of ECOFFinder were the same or similar to the CO_WT_ values obtained by NRI.

The CO_WT_ obtained in this study was compared with the results reported in CLSI [17]. The results showed that the CO_WT_ T values of *A. hydrophila* to erythromycin and florfenicol were consistent with the corresponding results of CLSI, and other CO_WT_ T values or CO_WT_ K values were similar to the corresponding result of CLSI. The maximum difference did not exceed two drug dilution gradients. The CO_WT_ obtained in this study was compared with the results reported by Baron et al. [12]. The CO_WT_ T values and CO_WT_ K values of *Aeromonas* spp. to trimethoprim–sulfamethoxazole and ceftazidime were exactly the same with the corresponding results reported by Baron et al. The CO_WT_ T values of *Aeromonas* spp. against erythromycin and enrofloxacin and *A. sobria* to trimethoprim–sulfamethoxazole and the CO_WT_ K values of *A. sobria* to ceftazidime were the same with the corresponding results reported by Baron et al. The rest of the results were similar to the corresponding results reported by Baron et al., except for the CO_WT_ T of *A. sobria* to enrofloxacin. The maximum difference did not exceed two drug dilution gradients. In conclusion, the CO_WT_ T values obtained in this study were closer to the corresponding results reported by CLSI and Baron et al. In addition, the results obtained in this study are the same or similar to those reported by CLSI and Baron et al., which indicates that the experimental methods referenced in this study are reliable and credible. On the other hand, it also shows that if a standard set of experimental methods for establishing breakpoints is used to establish CO_WT_ values, the CO_WT_ values obtained are generally the same or similar and are not greatly affected by regions.

In general, the MIC distribution of the drug against the strain presents a unimodal or bimodal distribution. For a typical bimodal distribution diagram, the right peak represents the MIC distribution of NWT strains, and the left peak represents the MIC distribution of WT strains. If the MIC distribution of a certain drug to the strain has an obvious bimodal distribution, CO_WT_ can be observed with the naked eye, that is, the upper limit of the MIC distribution of the WT strain. If the MIC distribution of the strain exhibits insignificant bimodal distribution or continuous multimodal characteristics, the fitting analysis of the MIC distributions can be performed with ECOFFinder or NRI to obtain more accurate CO_WT_ values [28]. In this study, the results showed that the MIC of enrofloxacin on *Aeromonas* spp. had a wide range and a multimodal distribution, indicating the existence of a higher proportion of enrofloxacin-resistant strains. *Aeromonas* spp. indeed had the highest resistance rate to enrofloxacin, as high as 61.5%/53.8%. The MICs of doxycycline and florfenicol against *Aeromonas* spp. were widely distributed and showed an insignificant bimodal distribution, indicating that a certain proportion of strains were resistant to doxycycline and florfenicol. The resistance rate of *Aeromonas* spp. to doxycycline and florfenicol was higher (9.3%/20.5% and 22.7%, respectively). This study found that the resistance rates of *Aeromonas* spp. collected from Xiashan reservoir and Liuchong river were low for most drugs compared with those of *Aeromonas* strains collected from aquaculture ponds, which may be related to the fact that these two natural water bodies have almost no drugs present. However, the resistance rates of these two water bodies to colistin or enrofloxacin were high. This was probably due to the surrounding industrial and agricultural production wastewater and domestic pollution. *Aeromonas* spp. in culture ponds had higher resistance rates to enrofloxacin, florfenicol, doxycycline, and trimethoprim–sulfamethoxazole. More importantly, enrofloxacin, doxycycline, and florfenicol are commonly used antibacterial drugs in aquaculture [22,26,27]. Therefore, more attention should be paid to the resistance changes of *Aeromonas* to these three kinds of drugs, and these antibacterial drugs should be rationally used in aquaculture practice to avoid the outbreak of drug-resistant strains caused by the abuse of antibiotics.

Compared with previous studies [12,33,34,35], the MIC_50_ or MIC_90_ of *Aeromonas* for the same drug in different studies was mostly different or quite different. This may be related to different human factors such as drug use, artificial breeding, and water pollution in different regions. However, the MIC_50_ and MIC_90_ of *Aeromonas* spp. and *A. sobria* to ceftazidime in this study was the same as the corresponding results of Baron et al. [12], and the resistance rates of this study and research of Baron et al. for *Aeromonas* spp. to ceftazidime were low, at 4.1% and 9.4%, respectively. This may be related to the fact that ceftazidime was less used in these two regions, and most of the existing *Aeromonas* were naturally sensitive to ceftazidime. The MIC_50_ and MIC_90_ of eight drugs against *Aeromonas* spp. obtained in this study can provide a theoretical reference for the usage and dosage of drugs in aquaculture.

The CLSI [36] document stipulates that more than 100 strains are required to establish the CO_WT_ values of bacteria of a species, whereas at least 500 bacteria need to be collected to establish the CO_WT_ values of a genus. However, the CLSI [37] document and Smith and Kronvall [38] suggested that only 30 strains may be required to statistically establish bacterial CO_WT_ values. The number of strains collected in this study met the above requirements.

The CO_WT_ values of *Aeromonas* for a variety of antimicrobial agents obtained in this study still need to be compared and integrated with data obtained by other laboratories to obtain internationally accepted CO_WT_ values for *Aeromonas* [39], which will facilitate the surveillance and epidemiological study of AMR in aquaculture environments.

## 5. Conclusions

In this study, the optimized micro-broth dilution method was used for the first determination of the MIC of eight drugs including trimethoprim–sulfamethoxazole, erythromycin, doxycycline, neomycin, colistin, florfenicol, enrofloxacin, and ceftazidime against aquatic *Aeromonas*. The tentative epidemiological cut-off values (CO_WT_ values) of these eight drugs against *Aeromonas* spp. were established by ECOFFinder and NRI methods, which were 0.25, 64/32, 4/2, 8, 4, 1, 0.062/0.125, and 0.5 μg/mL, respectively. Using these breakpoints as the standard, the proportion of NWT strains of *Aeromonas* spp. in different water bodies in China was analyzed. The results showed that *Aeromonas* spp. had a higher proportion of NWT strains against enrofloxacin, doxycycline, and florfenicol. Therefore, it is necessary to focus on the resistance changes of *Aeromonas* to these three drugs in the aquaculture environment and to conduct early monitoring of the resistance of *Aeromonas* to avoid the spread of drug-resistant strains.

## Figures and Tables

**Table 1:** Source and quantity distribution of 556 Aeromonas strains.

Sampling Location	Sampling Time	Water Type	Specimen Type	Host Species	No. of Samples	No. of Isolates
Wuhan, Hubei	2021	Fish Pond	Diseased fish	*Ctenopharyngodon idella*	3	6
				*Pelteobagrus fulvidraco*	2	3
			Healthy fish	*Ctenopharyngodon idella*	3	6
				*Pelteobagrus fulvidraco*	6	15
Jingzhou, Hubei	2020–2021	Fish Pond	Pond water	/	14	26
			Pond sediment	/	5	29
			Diseased fish	*Pelteobagrus fulvidraco*	118	267
				*Ctenopharyngodon idella*	3	17
				*Culter alburnus*	2	19
				*Hypophthalmichthys molitrix*	1	9
			Healthy fish	*Pelteobagrus fulvidraco*	1	2
Weifang, Shandong	2021	Reservoir	Diseased fish and healthy fish	*Hypophthalmichthys molitrix*	11	16
Bijie, Guizhou	2021	River	Healthy fish	*Hemicculter Leuciclus* (Basilewaky)	33	141

**Table 2:** MIC distributions of 556 Aeromonas strains against 8 drugs.

MIC (μg/mL)	0.016	0.031	0.062	0.125	0.25	0.5	1	2	4	8	16	32	64	128	256	>32	>64	>128	>256	HR (%)
TMP/SMZ			212	178	73	11	5	3		1			2				71			283 (50.9)
Erythromycin							1	7	89	199	150	51	14	5	10			14	16	30 (5.4)
Doxycycline				48	76	216	61	41	62	35	14	3								0 (0.0)
Neomycin						9	118	233	138	41	9	3	1	2				2		2 (0.4)
Colistin					4	43	250	123	62	44	10	10		5	2			1	2	3 (0.5)
Florfenicol				11	200	171	48	17	2	5	14	34	16	16	9			13		24 (4.3)
Enrofloxacin	21	178	15	43	89	89	46	15	4	24	16	12	4							99 (17.8)
Ceftazidime			64	264	145	60	15	2	1	1		1				3				59 (10.6)

MIC values equal to or lower than the lowest concentration tested are presented as the lowest concentration. The dilution range of the drug and the number of strain distribution contained in the white background was used for the formulation of CO_WT_ values, and HR (%) represents the number (percentage) of strains beyond the drug test range.

**Table 3:** The CO_WT_, MIC_50_, and MIC_90_ (μg/mL) and NWT (%) of *Aeromonas* spp.

Antimicrobial Agent	MIC_50_	MIC_90_	CO_WT_ k	CO_WT_ T	NWT (%)
Trimethoprim–sulfamethoxazole	0.125	>64	0.25 [0.25]	0.25 [0.25]	93 (16.7)
Erythromycin	8	64	64 [32]	32 [32]	45/59 (8.1/10.6)
Doxycycline	0.5	4	4	2	52/114 (9.4/20.5)
Neomycin	2	8	8	8	17 (3.1)
Colistin	1	8	4 [6.4]	4 [6.4]	74 (13.3)
Florfenicol	0.5	32	1 [4]	1 [2]	126 (22.7)
Enrofloxacin	0.25	8	0.062 [0.125]	0.125 [0.125]	342/299 (61.5/53.8)
Ceftazidime	0.125	0.5	0.5 [0.5]	0.5 [0.5]	23 (4.1)

CO_WT_ K/T represents the epidemiological cut-off values calculated by the Kronvall method or Turnidge method, the numbers in square brackets represent the breakpoints of the same drug studied by Baron et al. [12], no square brackets represent nothing can refer to. NWT (%) indicates the number (percentage) of NWT strains derived from the Kronvall method/Turnidge method, no forward slash represents the same results from the two methods.

**Table 4:** The CO_WT_, MIC_50_, and MIC_90_ (μg/mL) of four *Aeromonas* spp.

Antimicrobial Agent	*Aeromonas spp.* (*n* = 556)	*A. veronii* (*n* = 328)			*A. sobria* (*n* = 75)			*A. hydrophila* (*n* = 52)			*A. allosaccharophila* (*n* = 36)		
	CO_WT_ K/T	CO_WT_ K/T	MIC_50_	MIC_90_	CO_WT_ K/T	MIC_50_	MIC_90_	CO_WT_ K/T	MIC_50_	MIC_90_	CO_WT_ K/T	MIC_50_	MIC_90_
TMP/SMZ	0.25	0.25	0.125	>64	0.25 [0.5/0.25]	0.062	0.125	0.5	0.25	>64	1	0.25	>64
Erythromycin	64/32	32	8	128	16/32 [32/16]	8	16	128/64 (64)	16	32	32	8	256
Doxycycline	4/2	2	0.5	4	2	0.5	2	2	1	8	2/8	2	4
Neomycin	8	8	2	4	8	2	8	4	1	4	4/8	2	4
Colistin	4	4	2	8	4 [6.4]	1	2	4	1	32	8/4	1	2
Florfenicol	1	1	0.25	32	2 [1]	0.5	1	1/2 (2)	0.5	256	1/2	0.5	16
Enrofloxacin	0.062/0.125	0.062/0.125	0.25	8	0.062/1 [0.031/0.016]	0.25	0.5	0.062/0.125 (0.031)	0.25	2	8*/2	0.25	0.5
Ceftazidime	0.5	0.5	0.125	0.25	0.5 [0.5/0.25]	0.125	0.25	4*/2	0.25	0.5	0.5	0.25	1

*n* is the number, CO_WT_ K/T is the epidemiological cut-off values calculated by the Kronvall method or the Turnidge method, and only one value means the CO_WT_ values obtained by the two methods are the same. The numbers in square brackets represent the breakpoints of the same drug studied by Baron et al. [12], the numbers in parentheses represent the CLSI breakpoints, and the absence of square brackets or parentheses means the reference breakpoints are not available. The asterisk indicates SD exceeds the limit of the NRI program.

## Data Availability

Not applicable.
